# Erosion of the sella turcica and pituitary expansion secondary to polymicrobial brain abscesses: a case report

**DOI:** 10.1099/acmi.0.000270

**Published:** 2021-10-20

**Authors:** Brendan Ryu, Deepak Khatri, Avraham Zlochower, Stephen Maslak, Randy S. D’Amico

**Affiliations:** ^1^​ Department of Neurosurgery, Lenox Hill Hospital, Donald and Barbara Zucker School of Medicine at Hofstra/Northwell, New York, NY, USA; ^2^​ Department of Radiology, Lenox Hill Hospital, Donald and Barbara Zucker School of Medicine at Hofstra/Northwell, New York, NY, USA; ^3^​ Department of Internal Medicine, Division of Infectious Diseases, Lenox Hill Hospital, Donald and Barbara Zucker School of Medicine at Hofstra/Northwell, New York, NY, USA

**Keywords:** brain abscess, osteomyelitis, pituitary gland, sella turcica, SIADH

## Abstract

**Introduction:**

Brain abscesses can lead to a diverse array of complications, especially when they are polymicrobial in nature. Multiple underlying pathogens may present with a unique set of clinical symptoms which require an early identification and treatment. Skull base osteomyelitis with sellar floor erosion and pituitary involvement with SIADH are such rare complications of brain abscesses which have never been reported previously in the literature.

**Case Presentation:**

We report the case of an immunocompetent 38-year-old male with altered mental sensorium and left hemiparesis due to polymicrobial brain abscess which required surgical evacuation. The post-operative recovery was complicated by severe hyponatremia secondary to SIADH which was treated uneventfully. Radiological imaging demonstrated pituitary enlargement with herniation through an eroded sella turcica without active CSF leak. Patient responded well to the antibiotic therapy based on microbiological susceptibility testing with a complete resolution of the pituitary enlargement on radiological follow-up.

**Conclusion:**

Conservative treatment with targeted antibiotics can lead to the resolution of pituitary enlargement secondary to a brain abscess. However, a close clinical follow-up is required to look for a CSF leak considering the sellar floor erosion due to osteomyelitis.

## Introduction

Brain abscesses (BAs) are rare, focal collections of pus formed in the brain parenchyma with an incidence of 1–2 % in the western countries [1, 2]. Neurosurgical intervention is frequently required for the diagnosis and treatment of this condition. With the advent of image-guided biopsies, advanced microsurgical techniques, and targeted antibiotics, the mortality rate of patients with BAs significantly declined from 40–10 % over the past six decades [3]. Polymicrobial infections involving multiple bacterial species account for approximately 23% of the BAs and pose significant challenges in the diagnosis as well as the treatment of the disease.

We present a case of multifocal polymicrobial BAs occurring in an immunocompetent patient requiring a craniotomy for evacuation. Intraoperative cultures demonstrated polymicrobial growth with the predominance of *Fusobacterium nucleatum *, *Prevotella* spp*., Corynebacterium striatum, Corynebacterium minutissimum, Propionibacterium acnes,* and *Parvimonas micra *. Post-operative recovery was complicated by development of syndrome of antidiuretic hormone secretion (SIADH) with severe hyponatremia. MRI brain demonstrated a significant enlargement of the pituitary gland with herniation through the eroded floor of sella turcica as a result of the infection.

While clival, vertebral, and long-bone osteomyelitis due to *Fusobacterium* spp. and *Prevotella* spp. has been reported earlier, this marks the first description of skull base erosion with concomitant enlargement of pituitary gland secondary to a polymicrobial infection [4–7]. Here, we present this unique case along with a brief review of the pertinent literature.

## Case report

A 38-year-old male presented to our emergency department after being found unresponsive by his roommate. The patient had been experiencing severe headaches with painful neck stiffness, fatigue, myalgia, loss of appetite, and significant weight loss over 1 month which he had attributed to COVID-19 infection despite a negative testing. The patient was febrile (38.3 °C) with a stable blood pressure at the time of admission. On physical examination, weakness of the left upper and lower extremity (MRC grade 3/5) was observed. Cranial nerves and motor function on the right side were intact.

**Fig. 1.**
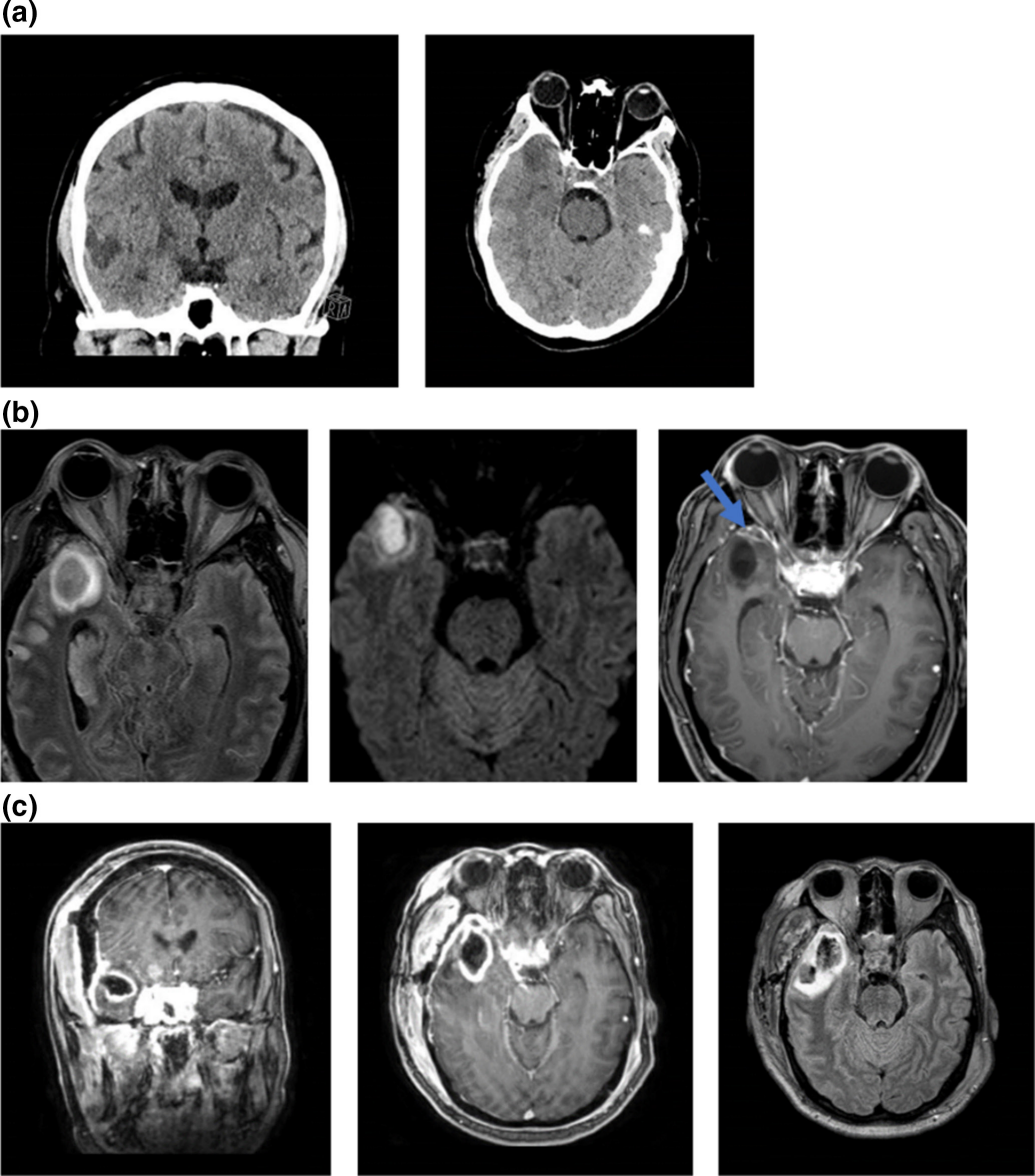
Pre- and post-operative neuroradiological studies identifying the brain abscess. (**a**) Axial (left) and coronal (right) non-contrast CT head demonstrate a hypodense 2.5 cm right anterior temporal lobe lesion. (**b**) Axial T2-FLAIR, DWI, and post-contrast T1 weighted images demonstrate a right anterior temporal cystic lesion with surrounding vasogenic oedema (left). There is central restricted diffusion (middle) and subtly peripheral enhancement (right, arrow) consistent with abscess. (**c**) Post-operative coronal and axial post-contrast T1 weighted images (left and middle) and axial T2-FLAIR images (right) demonstrate a post-operative hematoma in the right temporal lobe with persistent peripheral enhancement due to possible residual abscess.

Routine bloodwork demonstrated a leukocytosis of 14.3×10^3^ ul^−1^ (80 % neutrophils), elevated lactate level of 2.9 mmol l^−1^, erythrocyte sedimentation rate of 90 mm hr^−1^, C-reactive protein of 7.84 mg dl^−1^ and ALT/AST of 219/70 IU l^−1^. A sepsis protocol was initiated with fluid resuscitation and peripheral blood, urine, and cerebrospinal fluid (CSF) collection for the cultures. Broad spectrum intravenous antibiotics, vancomycin 1.5 g q8h and ceftriaxone 2 g q12h, as well as acyclovir 750 mg q8h were started for the empiric meningitis coverage and the patient was transferred under intensive care unit (ICU) care. Computed tomography (CT) of the head and magnetic resonance imaging (MRI) of the brain revealed a 2.5 cm in size, large cystic lesion located in the right anterior temporal lobe with surrounding oedema involving the anterior and mid temporal region, extending to the external capsule in its entirety and portions of the mid and posterior internal capsule, and a midline shift of 5 mm towards the left side. The cystic lesion demonstrated peripheral enhancement along with restricted diffusion on diffusion weighted imaging (DWI) suggesting a diagnosis of an abscess (Fig. 1a, b). Additional abscesses were also noted in the right temporal lobe, basal ganglia, and the internal capsule. Lumbar puncture and CSF analysis revealed a substantial pleocytosis of 375 ul^−1^ with 75 % polymorphonuclear leucocytes, low glucose (21 mg dl^−1^), and high protein levels (65 mg dl^−1^) suggestive of a pyogenic infective aetiology. However, the cultures from blood, urine, and CSF samples did not grow any organisms. HIV, HSV, acid-fast bacilli, *Toxoplasma gondii*, *Mycobacterium tuberculosis *, and fungal etiologies were ruled out.

The patient’s clinical condition worsened despite broad spectrum antibiotics, and he developed a progressively altered mental status over the next couple days. Repeat CT imaging showed worsening cerebral oedema and the patient was taken for a right temporal craniotomy and abscess drainage. Histopathological examination revealed necrotic cells with inflammation suggesting a fibrinopurulent abscess with no evidence of tumour cells. Antibiotics were subsequently transitioned to intravenous (IV) metronidazole (750 mg q8h), ceftriaxone (2 g q12h) and vancomycin (1.5 mg q8h). The patient was also treated with a routine corticosteroid taper postoperatively (dexamethasone 4 g q12h for 3 days, 3 g q12h for 3 days, 2 g q12h for 3 days, 1 g q12h for 3 days, 1 mg q24h for 3 days).

After the surgical evacuation of the abscess, the patient responded well to the antibiotic treatment with improvement in his clinical examination and normalization of the leucocyte count (10.2×10^3^ ul^−1^). Post-operative MRI on day 3 demonstrated an evacuation of the right temporal abscess with no foci of contrast enhancement. However, the vasogenic perilesional oedema was persistent. (Fig. 1c). The patient’s neurologic examination remained similar, which may be attributed to the involvement of the basal ganglia and persistent right temporal oedema.

**Fig. 2.**
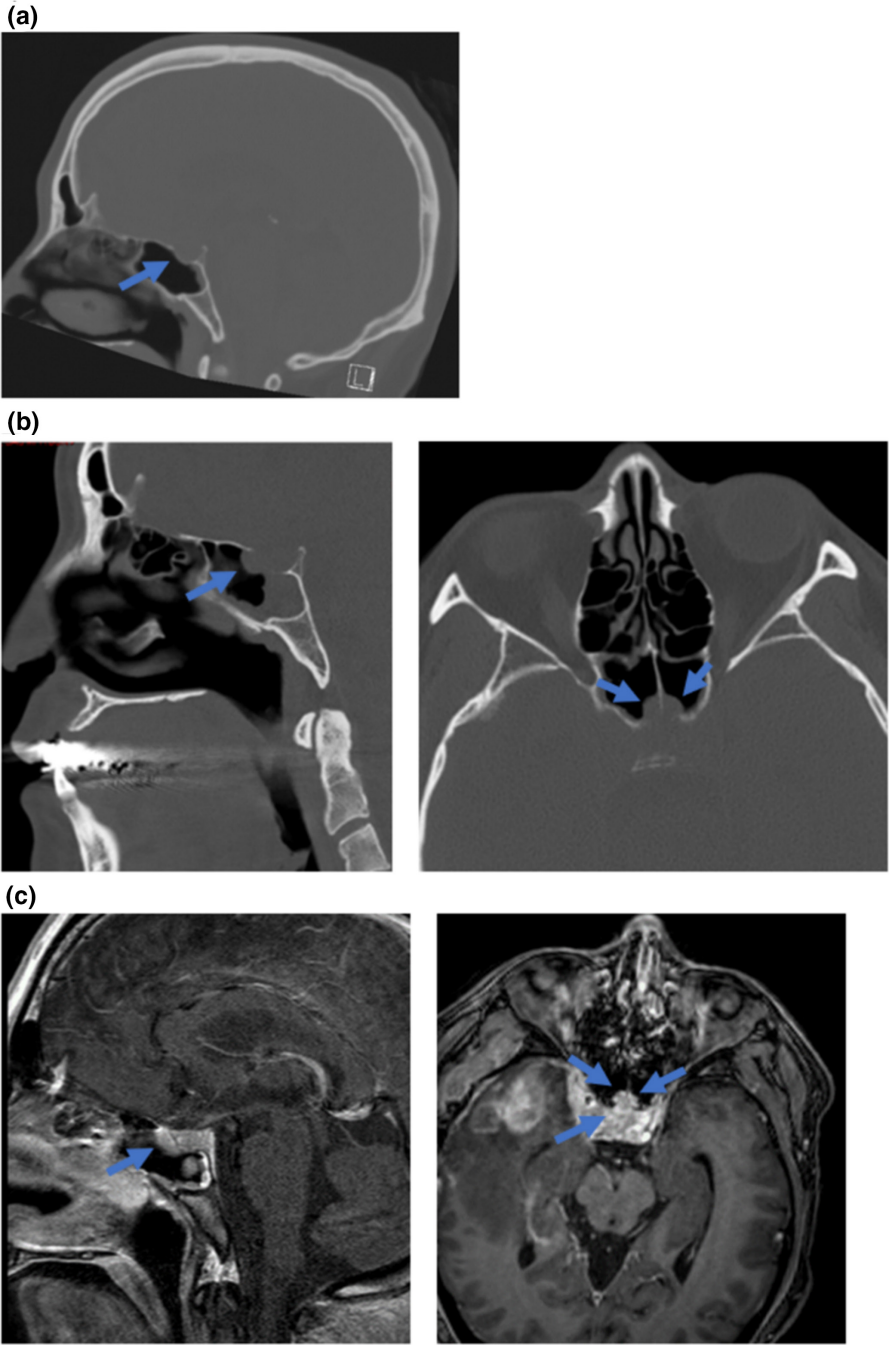
Pituitary expansion and erosion of the sella turcica. (**a**) Pre-operative CT in bone window demonstrates an intact anterior sella wall without evidence of dehiscence and pituitary herniation. (**b**) Post-operative day 6 CT sinus in bone window demonstrates a defect in the anterior wall of the sella with herniation of sellar contents into the bilateral sphenoid sinus. (**c**) Post-operative day 7 T1-weighted MRI images demonstrates a heterogeneously enhancing pituitary tissue herniating through anterior sellar defect into bilateral sphenoid sinuses.

On the fourth post-operative day, the patient developed acute hyponatremia (129mEq l^−1^). Further work-up demonstrated an elevated urinary osmolality (753mOsm kg^−1^) and urinary sodium suggesting the diagnosis of SIADH. The CT of the head obtained at this time (post-operative day 6) demonstrated a new bony defect at the central skull base involving the anterior wall of the sella with herniation of its contents into the sphenoid sinus (Fig. 2). Next day, MRI to visualize the sella turcica was performed which demonstrated the herniation of an enlarged pituitary mass through a defect in the sellar floor with extension into the sphenoid sinus. The pituitary mass lesion showed heterogeneous signal on T1 and T2 weighted images and contrast enhancement in the gland suggesting infection with no identification of a drainable abscess (Fig. 2). The patient was fluid restricted with gradual improvement in his sodium levels to normonatremia.

**Fig. 3.**
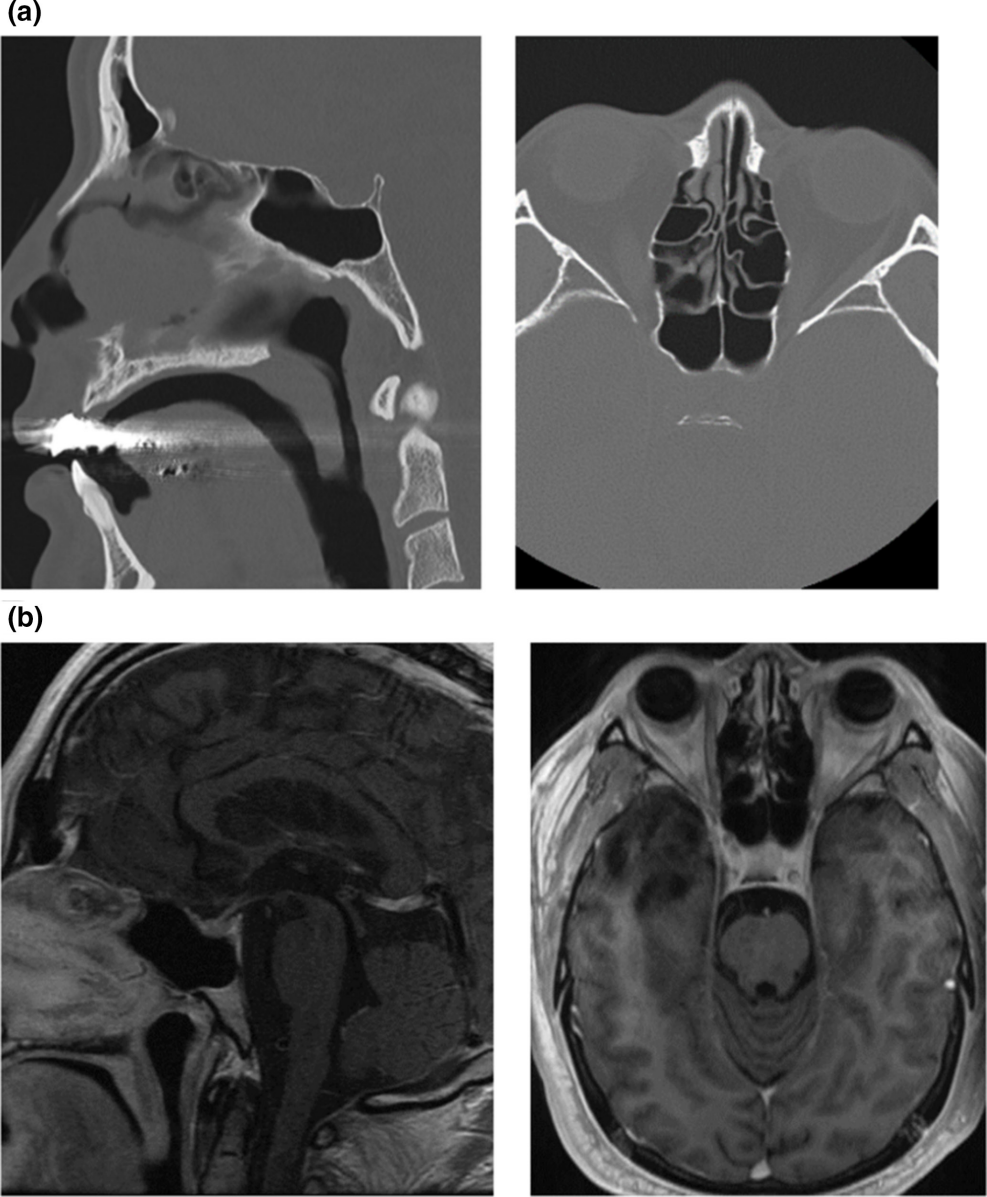
Resolution of the pituitary expansion and erosion of the sella turcica. (**a**) CT sinus bone windows demonstrates resolution of herniation of pituitary tissue and repair of sellar defect. (**b**) Post-contrast T1-weighted MRI demonstrates resolution of pituitary tissue herniation with normal enhancement pattern.

Pathogens from the specimen were identified via matrix assisted laser desorption ionization-time of flight mass spectrometry (MALDI-TOF MS), and *Fusobacterium nucleatum, Corynebacterium striatum*, *Corynebacterium minutissimum, and Propionibacterium acnes* were grown on culture for antibiotic susceptibility testing done on Etest. *Parvimonas micra* and *Prevotella* spp. did not grow on culture for antibiotic susceptibility testing. Antibiotic susceptibility testing guided the transition to a final regimen of IV penicillin G (4 million units q4h), oral metronidazole (750 mg q8h), and intravenous ceftriaxone (2 g q12h) for a 6 week course. Ceftriaxone was empirically prescribed before the pathogens were identified to cover Gram-negative bacteria. Furthermore, it was not discontinued in the final regimen since the infectious disease physicians were uncomfortable with the coverage by metronidazole and penicillin. Metronidazole, which was prescribed to cover *Fusobacterium nucleatum *, was shown to have a minimum inhibitory concentration (MIC) of 0.016. Penicillin, used to cover *Corynebacterium striatum *, *Corynebacterium minutissimum, and Propionibacterium acnes,* was shown to have a MIC of 0.064, 0.38, and 0.064, respectively. The patient continued to improve and was discharged with 3 months of antibiotics and a steroid taper. MRI at 6 month follow-up showed stable recovery from the procedure. Interestingly, pituitary enlargement seen previously was no longer visualized and the sella appeared grossly unremarkable (Fig. 3). Our patient was watched closely for presence of a potential CSF leak with consultation with the Otolaryngology department and the assessment showed no sign for CSF leak.

## Discussion

Brain abscesses are rare in healthy individuals without an ongoing infection; however, they are associated with a significant risk of neurological complications and death [3]. Symptoms are mostly non-specific. While a triad of fever, headache, and focal neurologic deficit has been classically described, it is only observed in approximately 20 % of cases at presentation. Other described symptoms include nausea, altered consciousness, seizures, and nuchal rigidity [3]. Monobacterial BAs contribute to the majority (77 %) of BAs and are most commonly due to *Streptococcus* spp. and *Staphylococcus* spp. Polymicrobial BAs are thought to arise in approximately 23 % of cases [3]. Common risk factors and predisposing conditions include otitis, sinusitis, heart diseases, trauma, pulmonary disease, odontogenic and immunocompromised status. Parasitic and fungal etiologies for BA are very rare, comprising less than 1 % of all BA etiologies; fungal brain abscesses most likely suggest an immunocompromised status for the patient [3]. However, mortality for patients with BA secondary to a fungal infection has been shown to be over 80 % [8].

We discussed the case of a young, immunocompetent male who presented initially with a polymicrobial BAs that progressed despite antibiotic treatment to involve the pituitary gland, as well as the bony skull base. Subsequently, the patient developed SIADH and became hyponatremic secondary to pituitary gland involvement. Neurosurgical patients admitted with intracranial tumours and in patients undergoing pituitary surgery, hyponatremia occurs in between 10 and 20 % of patients [9]. Bony erosion elsewhere with osteomyelitis secondary to the organisms involved in this case has been recognized earlier, however, involvement of the sella and pituitary has been rarely reported [10]. The patient underwent biopsy and evacuation of the accessible temporal abscess and was ultimately managed with symptomatic treatment and appropriate antibiotics based on the microbiological susceptibilities with excellent recovery. These results provide support that in the rare setting of brain abscess with pituitary involvement and osteomyelitic erosion of the sella without a CSF fistula, medical management can be maintained with great success.

While rare in immunocompetent patients, recent dental work could be a potential source of this reported infection. Microbiological cultures in this case grew *Fusobacterium nucleatum *, *Prevotella* spp., *Corynebacterium striatum *, *Corynebacterium minutissimum, Propionibacterium acnes,* and *Parvimonas micra. F. nucleatum* are anaerobic Gram-negative bacilli that are ubiquitously present in the human oral cavity with potential reservoirs in the gastrointestinal tract and other parts of the body including the heart, liver, and the brain [11]. It is most commonly associated with oral and periodontal pathologies and has been implicated in BA, especially when associated with Lemierre’s syndrome [12–14]. *Prevotella* spp. are anaerobic Gram-negative bacilli commonly identified in oral, vaginal, and gut microbiota and a known culprit causing BA in patients with a history of dental procedures [6, 15, 16]. *Corynebacterium* spp. are aerobic Gram-positive bacilli that are part of the resident skin flora. In particular, *C. striatum* commonly causes lower respiratory tract and central line infections. *C. striatum* has rarely been reported to cause meningitis and suspected BAs [3, 17]. *C. minutissimum* has mainly been known as the causative agent of erythrasma and has only been reported once as an infecting pseudomeningocele [18]. *P. acnes* are anaerobic Gram-positive bacilli that are part of the normal flora of the skin, nasopharynx, oral cavity, and gastrointestinal and genitourinary tracts. Due to its prevalence in the scalp flora, *P. acnes* has been linked post-neurosurgery infections such as BAs and subdural and epidural empyemas [19]. *P. micra* are anaerobic Gram-positive cocci and a commensal organism of the oral cavity and gastrointestinal tract. They have been reported to cause odontogenic BAs; however, the pathogen is usually not identified on standard bacterial cultures and require a 16S rRNA gene sequencing analysis [20]. In the case of the patient, MALDI-TOF MS successfully identified *P. micra *.

Treatment of BAs remains surgical drainage (when appropriate) and treatment with antibiotics as per the susceptibility testing. This case is unique in that our patient developed SIADH due to pituitary involvement and bony erosion of the sella suggesting a component of skull base osteomyelitis. Pituitary gland expansion has not previously been described in the setting of any of the pathogens identified in this patient. Radiological findings in our patient did not demonstrate a drainable abscess in the pituitary gland, but there was a high suspicion for an underlying infection due to heterogenous signal and enhancement in the gland.

While rare, pituitary abscesses (PA) have been described and account for less than 1 % of all pituitary lesions. They have been described secondary to *Staphylococcus* spp, *Streptococcus* spp, *Neisseria* spp, and *Escherichia coli* infections [21, 22]. About 70 % of the pituitary abscesses arise *de novo*, but may complicate a pre-existing lesion, such as a pituitary adenoma [22]. While it is possible that pituitary enlargement can be explained by an inflammatory reaction secondary to pituitary infarction, new bony erosion of the sella in the setting of pituitary expansion argues more for an infective aetiology. Endocrine abnormalities, such as central diabetes insipidus and hyperprolactinemia, have been associated with PA patients [21, 22]. However, SIADH is a rare complication due to pituitary inflammation in the setting of multifocal polymicrobial BA. There has been one reported case of clival osteomyelitis due to *F. nucleatum* and *Campylobacter rectus* following a tooth extraction [7]. Interestingly, *F. necrophorum,* is more commonly linked to Lemierre’s syndrome and has been reported to cause clival osteomyelitis [23]. In addition, *Fusobacterium* spp. has been isolated in animals such as cattle, dogs, and cats presenting with signs of central nervous system infection such as mental depression and focal neurologic deficits [24]. One case of a cat showed sellar osteomyelitis and pituitary exudates due to *Fusobacterium* spp., *Bacteroides* spp., and *Eubacterium* spp [24]. Otherwise, sellar osteomyelitis along with pituitary expansion due to *F. nucleatum, Corynebacterium* spp*., Prevotella* spp., *Propionibacterium acnes *, and *Parvimonas micra* infection has never been reported.

## Conclusion

Although rare in immunocompetent individuals, polymicrobial brain abscess can be challenging to diagnose and treat. Surgical decompression with early identification of the responsible microbes and targeted antibiotic treatment based on susceptibility forms the mainstay of treatment. Rare complications such as skull base osteomyelitis and involvement of the pituitary gland with subsequent SIADH may adversely affect the recovery and clinical outcome if not identified and treated appropriately.
